# Tracer Techniques in Ophthalmology: Ocular Applications and Systemic Connections

**DOI:** 10.3390/diagnostics16111608

**Published:** 2026-05-25

**Authors:** Xinxin Ye, Liting Zhao, Xiaodi Zhou, Wenyi Wu, Ying Lu, Yuanjun Li, Yewei Yin, Tu Hu, Dan Wen

**Affiliations:** 1 Eye Center of Xiangya Hospital, Central South University, Changsha 410008, China; yexinxin202602@163.com (X.Y.); z17873154164@163.com (L.Z.); zhouxd0901@csu.edu.cn (X.Z.); 15200811001@163.com (W.W.); lyacxlx@outlook.com (Y.L.); yywei1990@csu.edu.cn (Y.Y.);; 2 Hunan Key Laboratory of Ophthalmology, Changsha 410008, China; 3 National Clinical Research Center for Geriatirc Diseases, Xiangya Hospital, Central South University, Changsha 410008, China; 4 National Clinical Key Specialty of Ophthalmology, Changsha 410008, China

**Keywords:** tracer techniques, ocular imaging, theranostics, ocular system connections

## Abstract

Tracer techniques have emerged as pivotal tools in ophthalmology, offering unprecedented capabilities for visualizing and quantifying complex biological processes within the eye. These techniques—spanning optical, isotopic, and metal-based tracers—have significantly enhanced our ability to detect and monitor ocular diseases, from early-stage pathologies to therapeutic responses. By providing molecular-level specificity, improved signal sensitivity, and real-time dynamic imaging, tracers enable precise analysis of ocular fluid dynamics, retinal and vascular abnormalities, and neural connections between the eye and brain. Furthermore, these technologies are advancing our understanding of the systemic connections between the eye and other organs, such as the brain, thyroid, and lymphatic systems. Tracers are helping to uncover new pathways for understanding these relationships and their impact on both ocular and systemic diseases. Despite challenges in clinical translation, biosafety, and specificity, advances in tracer design, particularly at the nanoscale, are driving the development of multimodal imaging platforms. As these technologies continue to evolve, they will increasingly support the development of tailored treatment regimens, improve early detection, and facilitate the monitoring of therapeutic outcomes.

## 1. Introduction

The clinical management of ocular diseases faces unique challenges stemming from ocular-specific characteristics: the organ’s vulnerable position, intricate anatomical structures, and unique physiological barriers that restrict the passage of lipophobic drugs, and hydrophilic macromolecules [1].

Clinical application of ophthalmic imaging modalities is limited in ophthalmology [2,3,4]. The diagnostic reliability of optic disc photography is influenced by subjective assessment [5,6]. Ocular tracers employ specific labels to enable visualization and quantitative analysis of biomolecules, cells, or tissues, overcoming the limitations of conventional structural imaging to dynamically monitor ocular hemodynamics, molecular transport, and cellular migration [7,8,9]. The integration of tracer techniques with multimodal imaging platforms, including fluorescence microscopy, optical coherence tomography angiography (OCTA), and magnetic resonance imaging (MRI), provides high spatiotemporal resolution for quantitative analysis, particularly for investigating ocular disease pathogenesis and therapeutic responses [10].

Tracer-based molecular imaging in ophthalmology offers unique advantages in clinical practice [11,12,13,14]. The monitoring efficiency has been significantly improved, enabling the early detection of ocular diseases such as glaucoma and diabetic retinopathy, even before clinical symptoms manifest [15]. Additionally, ocular lesions are the initial manifestations of multiple systemic diseases, including Behçet’s syndrome, multiple sclerosis, and Sjögren’s syndrome. As a result, the eye is recognized as a window to systematic health [16,17]. The early detection of ocular lesions is beneficial to the management of multiple systematic diseases [18,19,20].

Tracer techniques have become indispensable tools in ophthalmic practice, facilitating research, diagnosis, and therapeutic interventions. However, there is no comprehensive review focusing on the clinical applications of ocular tracers. This review categorizes the principles and advantages of current technologies while evaluating their roles in early diagnosis, prognostic prediction, and treatment response assessment. Furthermore, we explore their potential in personalized therapeutic strategy, advancing the translation of precision ophthalmology into clinical practice.

## 2. Tracer Techniques in Ophthalmology

### 2.1. Optical Tracer Techniques

The transparent ocular media—including the cornea, aqueous humor, lens, and vitreous body—exhibit high light transmittance, which enables efficient transmission and detection of tracer signals [21]. Optical tracer techniques utilize light–matter interactions to label, detect, and image target fluids, cells, or tissues. Based on the type of signal detected, they can be broadly categorized into two main branches: fluorescent tracers, which rely on emitted photons, and photoacoustic tracers, which rely on acoustic waves.

#### 2.1.1. Fluorescent Tracers

Conventional fluorescent imaging contains intravenous injection of low-molecular-weight dyes, achieving the real-time assessment of vascular perfusion and leakage [22]. Among these, fluorescein sodium and indocyanine green (ICG) are the most used clinical tracers, powering fluorescein angiography (FA) and indocyanine green angiography (ICGA), respectively. Despite their shared role in angiography, their distinct physicochemical properties lead to different clinical applications. Sodium fluorescein is a water-soluble organic compound with a molecular weight of 376 Da. Because of its low molecular weight, it can leak through the fenestrated endothelium of the choriocapillaris, thus limiting the application in choroidal vessel imaging. As a result, FFA is primarily used for retinal vessel imaging rather than choroidal vessel imaging. Sodium fluorescein emits strong fluorescence under conditions of nearly physiological pH (7.4), which matches the blood environment. FFA is a diagnostic tool for detecting choroidal and retinal leakage in ocular pathologies [23]. The molecular weight of ICG is 775 Da, which is greater than sodium fluorescein, and ICG preferentially binds to lipoproteins. The large size of this complex prevents it from leaking out of the choriocapillaris. A comparison of FFA and ICGA in ophthalmic diagnostics is given in Table 1.

**Figure 1:**
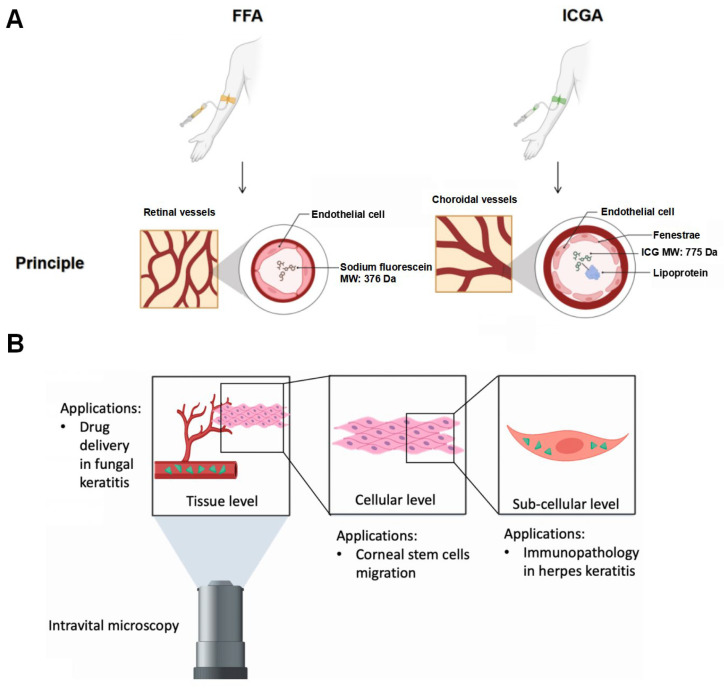
Mechanism of fluorescent tracers. (**A**) FFA is mainly used for retinal vessel imaging. ICGA prefers to bind to lipoprotein and is used for choroidal vessel imaging. (**B**) Two-photon microscopy is applied to ocular tissues and physiological and pathological process imaging. (**B**) reproduced from Reference [44], with the permission of MDPI, under the Creative Commons Attribution License. FFA: fluorescein fundus angiography; ICGA: indocyanine green angiography; MDPI: Multidisciplinary Digital Publishing Institute.

Cellular fluorescent tracers enable real-time monitoring of microenvironments such as polarity, pH, and viscosity. They exhibit alterations in emission wavelength and fluorescence intensity in response to microenvironment changes, enabling continuous tracking of transplantation, migration, division, fusion, and lysis [40]. For example, a combination of light sheet fluorescence microscopy with a clearing technique offers new insight into 3D eyeball imaging [41]. This method monitors complex structures such as retinal capillaries and anterior ciliary vessels with high spatial resolution, which is useful for ocular anatomical pathology research. Moreover, this high spatial resolution enables in-depth observation of ocular microstructures and pathological changes, providing new tools for early diagnosis and monitoring of ophthalmic and neurodegenerative diseases [42,43]. In the investigation of ocular immunology, multiphoton fluorescence microscopy (MPM) is widely used for in vivo tissue imaging, especially in the study of cellular and subcellular processes within ocular tissues (Figure 1) [44]. MPM penetrates deep into ocular structures and offers high-solution imaging, exhibiting advantages in research on infectious keratitis and corneal immunology. This technique can track the migration and interactions of immune cells, which provides a deeper understanding of ocular immune responses. Furthermore, unlike conventional sodium fluorescein staining, the combination of fluorescence microscopy and impression cytology not only monitors dynamic changes in cell layers but also provides more detailed cellular behavior information. This is significant for studying the migration, division, and interactions of corneal epithelial cells [45].

Fluorescent tracers conjugated with drug molecules or delivery vehicles enable the tracking of the in vivo fate of drugs non-invasively and in real time. The fluorescent dye DIO is used to label lipid nanocapsules (LNCs) and liposomes, exploring the potential of LNCs as intravitreal injection carriers [46]. DIO exhibits slow leakage rate from the carrier, reflecting the carrier behavior accurately. Research has shown that approximately 48% of liposomes’ signal distributes in the retinal area, while about 40% of the LNCs’ signal distributes in ciliary body. This distribution difference is attributed to the surface properties and size of LNCs, which enhance uptake by ciliary body epithelial cells. This finding provides experimental evidence for the design of ciliary body-targeted drug delivery systems in IP reduction treatment. A novel optimized LNC that loads with travoprost has been shown to boost the bioavailability of the drug threefold in glaucoma therapy [46]. Coumarin 6 (C6) has been used to non-invasively and quantitatively track from the ocular surface to intraocular tissues in animal models. C6 has successfully proved clinical validity of new drug delivery systems that are capable of loading sunitinib and sirolimus to treat AMD, DR, and uveitis [47].

Fluorescent tracer techniques are capable of high-throughput screening of promising delivery systems in ocular therapies. Drug delivery systems (DDSs) such as nanoparticles and liposomes have bioadhesive properties that greatly overcome physiological clearance barriers, including tear turnover, nasolacrimal drainage, and reflective blink. A new nanosystem has been designed to address low bioavailability, composed of montmorillonite, hyaluronic acid, and chitosan [48]. To prove bioavailability, the active drug is replaced with sodium fluorescein and then fluorescent nanoparticles are prepared in an identical process. The retention time of signal from the nanoparticle group is about 85 min, a 4.6-fold extension compared to normal drugs, under a real-time record of fluorescence intensity on the corneal surface using a slit lamp. Ocular fluorescent tracers are beneficial in offering safer, more efficient, and non-invasive therapies for blinding eye diseases.

#### 2.1.2. Photoacoustic Tracers

**Figure 2:**
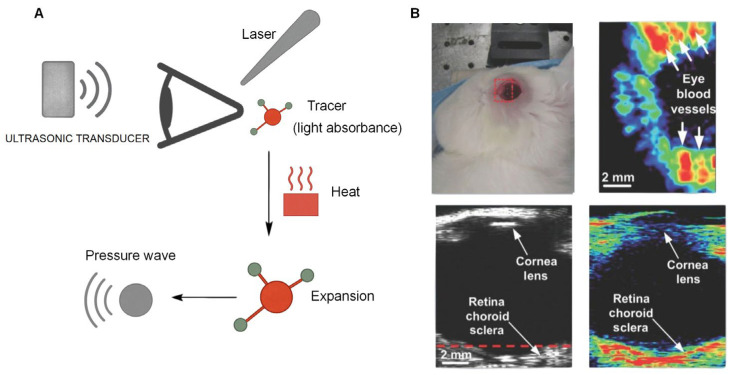
Mechanism and application of photoacoustic tracers. (**A**) A photoacoustic tracer is irradiated by a laser beam, absorbs optical energy, and converts it into heat, causing rapid thermal expansion and a rise in pressure able to be detected by ultrasonic transducers. (**B**) Photographic, horizontal photoacoustic, vertical ultrasound, and vertical photoacoustic images of the rabbit eye. (**B**) Reproduced with permission from [60], Optica Publishing Group.

Photoacoustic imaging (PAI) is a non-ionizing, combined modality. Unlike conventional optical imaging, PAI overcomes the limitations of light scattering, achieving a penetration depth of up to a few centimeters in biological tissue [49]. It irradiates the photoacoustic tracer with a laser beam. The tracer absorbs optical energy and converts it into heat, causing rapid thermal expansion and a rise in pressure [50,51]. Finally, it can be detected by ultrasonic transducers [52]. PAI bypasses light scattering and is suitable for deep tissue imaging, especially for ocular imaging, because of optical transparency in ocular media such as the cornea, lens, and vitreous body [53]. In ocular imaging, hemoglobin [54], which is located in microvessels of the retina, conjunctiva, and tumors, and melanin, which exists in the uvea and pigmented cancers, are essential light-absorbing molecules [55]. With the development of contrast agents, such as organic small molecules, nanobodies [56], and reporter genes [57,58], PAI has improved contrast and resolution. For example, photoacoustic tracers distinguish choroidal neovascularization (CNV) from adjacent normal microvasculature within the choroid. This technique bypasses optical scattering barriers and achieves a resolution of up to 4.1 μm, overcoming the limitations of conventional methods that suffer from insufficient resolution (Figure 2) [59,60].

### 2.2. Isotope-Based Tracer Techniques

Isotopes are atoms of the same element that contain different numbers of neutrons, but exhibit similar chemical properties [61]. Based on their ability to undergo radioactive decay, they are classified as either radioactive or stable isotopes. Through physical decay, radioisotopes are used for quantitative analysis, even capable of detecting trace quantities as low as 10^−18^ grams [62]. Stable isotopes do not undergo radioactive decay, but they can be detected by nuclear magnetic resonance (NMR) or mass spectrometry (MS) [63]. Unlike optical tracers, isotope-based tracing is unaffected by tissue opacity, autofluorescence, or light scattering. Furthermore, the high spatial resolution allows for the localization of pathological processes in ocular microstructures, making isotopic tracing a useful tool in ophthalmology.

#### 2.2.1. Radioisotope Tracers

Radiopharmaceuticals form the cornerstone of nuclear medicine, contributing to early diagnosis, treatment response evaluation, and radiotherapy [64]. Based on the type of radiation emitted, they can be classified into those that emit positrons and those emit γ-rays. They differ in physical properties, such as penetration depth and ionization capacity, which in turn dictate their respective medical applications.

***Positron-emitting isotopes.*** Positron-emitting isotopes are used in positron-emission tomography (PET) imaging [65]. After injection, biomolecules labeled with radionuclides circulate systemically and accumulate in target tissues [66]. ^18^F-fluorodeoxyglucose (FDG) is transported into cells through glucose transporters 1 and 3 (GLUT-1 and GLUT-3), which are often overexpressed in malignant and inflammatory cells owing to the heightened glucose metabolic activity [67]. After cellular uptake, FDG is phosphorylated by hexokinase to form FDG-6-phosphate. Because this metabolite cannot be further processed by glycolytic enzymes, it becomes trapped and accumulates within the cell. Simultaneously, the positrons emitted during the radioactive decay of the fluorine-18 isotope (^18^F) are detected by the PET scanner. By reconstructing the three-dimensional distribution of the radiotracer in vivo, PET enables the visualization and quantification of key biochemical processes such as glucose metabolism, blood perfusion, and receptor density [68,69,70]. Preclinical studies in animal models reveal that PET tracers such as [^18^F]DPA-714 and ^18^F-FDG sensitively detect early and significant inflammatory signals in both blue light-induced retinal degeneration and endotoxin-induced uveitis models [71,72]. Mechanistically, [^18^F]DPA-714 targets an mitochondrial 18 kDa translocator protein (TSPO) expressed on activated microglia in the retina, which is specifically activated by blue light damage, a process simulating photoreceptor degeneration in early-stage AMD and glaucoma-related neuroinflammation [73].

**Figure 3:**
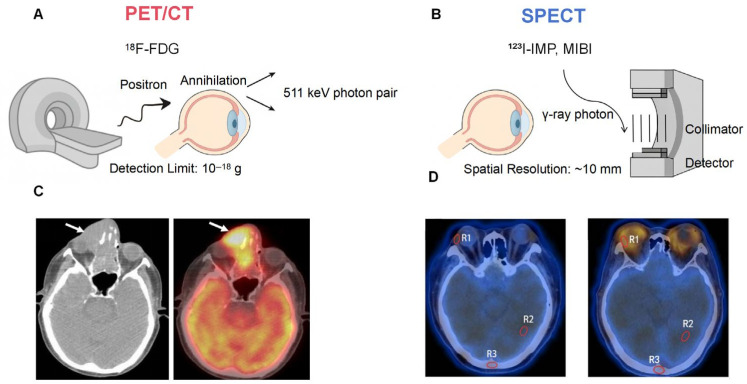
Mechanism and application of radioisotope tracers. (**A**) Imaging mechanism of ^18^F-FDG PET/CT. (**B**) Imaging mechanism of ^123^I-IMP and MIBI SPECT. (**C**) Fused ^18^F-FDG PET-CT coronal and axial images showing orbital lymphoma after surgery. (**D**) SPECT/CT imaging in inactive and active GO patients (R1: lacrimal gland, R2: occipital lobe brain region, R3: occipital region). (**C**) reproduced from Reference [68] under the Creative Commons Attribution-NonCommercial-NoDerivatives 4.0 International License, published by International Journal of Oncology. (**D**) reproduced from Ref. [78], Copyright © 2022, under the Creative Commons Attribution-NonCommercial 4.0 International License. ^18^F-FDG: 18-fluorodeoxyglucose; PET/CT: positron-emission tomography/computed tomography; ^123^I-IMP: 123-iodine-labeled iodophenyl-pentadecanoic acid; MIBI: methoxyisobutylisonitrile; SPECT: single-photon-emission computed tomography; GO: Graves’s orbitopathy; CT: computed tomography.

***γ-Emitting isotopes.*** Single-photon-emission computed tomography (SPECT) provides both quantitative and functional tracking capabilities [74]. In contrast to positron-emitting isotopes used in PET, γ-emitting isotopes employed in SPECT emit single γ-ray photons. A collimator is positioned in front of the detector to spatially select photons based on their direction of travel. Only photons traveling parallel to the collimator holes are detected, while others are absorbed. As a result, the spatial resolution of SPECT is lower than that of PET. To improve its spatial resolution, collimators with smaller and longer channels can be used. For example, systems equipped with parallel-hole collimators typically achieve a resolution of approximately 10 mm [75]. ^99m^Tc-sestamibi (MIBI) is a complex formed by radionuclide ^99m^Tc and the organic ligand sestamibi. As a metal ion, ^99m^Tc requires chelation with ligands to become stable. Sestamibi is a lipophilic isonitrile ligand with several isonitrile groups, which enable it to bind to ^99m^Tc tightly and cross the cell membranes, thus accumulating in the mitochondria. The pinhole collimator is used in the imaging of ^99m^Tc-MIBI and has been proved to have relatively higher resolution in pathological parathyroid glands and ocular malignant lesions [76]. Specifically, MIBI imaging can detect ocular malignant lesions ranging from 9.5 to 12 mm in diameter [77]. However, this limited resolution hinders the identification of millimeter-scale lesions such as DR and small retinoblastoma. The mechanism and application of radioisotope tracers are shown in Figure 3.

Theranostics integrates radioisotope tracers with targeted radionuclide therapy, thereby achieving precision medicine [7]. Radioactive tracers conjugated with specific drugs realize targeted, precision, and local therapy and reduce the toxic effects on nearby tissues. Therapeutic radionuclides emit cell-killing beta or alpha particles. The direct application of theranostics in ophthalmology is in the early stage, far less mature than in oncology. Future application in ocular tumors such as uveal melanoma, intraocular lymphoma, and retinoblastoma is promising [79].

#### 2.2.2. Stable Isotope Tracers

Nowadays, there are increasing health concerns over radiopharmaceuticals, making non-radioactive stable isotopes a more suitable choice for long-term tracing and in vivo studies. Through introducing nutrients labeled with tracers into the body, this technique can be used to study metabolism alterations, uncovering the pathogenesis of diseases.

**Figure 4:**
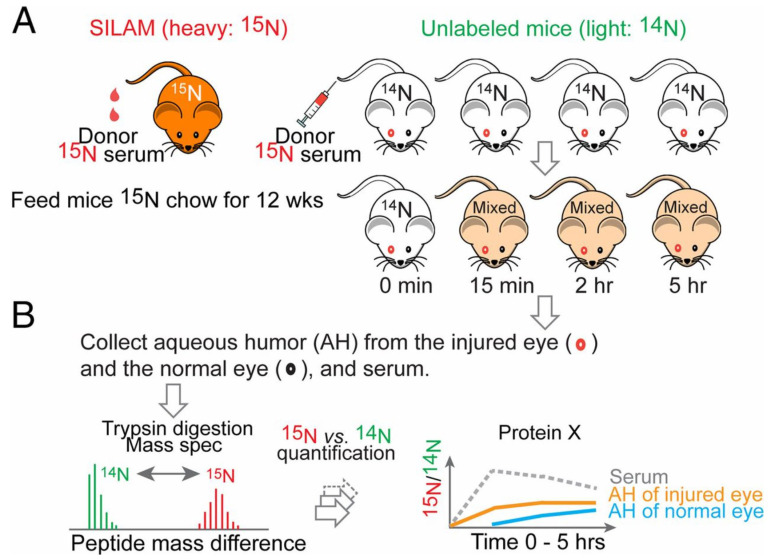
Mechanism and application of stable isotope tracers. Workflow for tracing plasma protein kinetics into the aqueous humor using ^15^N-labeled mouse serum. (**A**) ^15^N-labeled serum, obtained from SILAM donor mice, was injected (300 μL) into the tail vein of ^14^N recipient mice. (**B**) The entry of plasma proteins was quantified by LC-MS/MS, measuring the ^15^N/^14^N ratio for individual proteins in the aqueous humor. Reproduced from Ref. [84], with permission from the National Academy of Sciences. ^15^N: nitrogen 15; SILAM: stable isotope labeling by amino acids in mammals; ^14^N: nitrogen 14; LC-MS/MS: liquid chromatography–mass spectrometry/mass spectrometry.

The retina exhibits one of the highest energy demands in the human body. Photoreceptor (PR) cells in particular display a mass-specific oxygen consumption rate that exceeds even that of the brain. This energy supports phototransduction, maintenance of dark current, synaptic transmission, and the renewal of proteins and photopigments—all critical for sustaining vision [80]. Stable isotope tracing is important for probing metabolic activity, as it allows quantitative and dynamic tracking of metabolites labeled with non-radioactive isotopes such as ^13^C-glucose and ^13^C-glutamine, thereby illuminating specific metabolic pathways and flux rates [81]. For example, ^13^C-glucose labeling experiments have been applied to investigate metabolic alterations in photoreceptors following the knockout of both HK2 and PKM2 genes [82]. Given that nearly 80% of glucose in the retina undergoes aerobic glycolysis, glucose represents a well-suited substrate for evaluating the metabolic state of PRs using stable isotope tracing [83]. Stable isotope tracers are also valuable for studying protein turnover and transport. In one study, ^15^N-labeled mouse serum was injected into recipient mice and LC-MS/MS used to quantify the ^15^N/^14^N ratio of individual proteins entering the aqueous humor (Figure 4) [84]. This approach allows dynamic tracking of plasma protein kinetics across the blood–aqueous barrier.

### 2.3. Metal-Based Tracers

Metal-based tracers represent a class of contrast and therapeutic agents that utilize metallic elements—such as gold and gadolinium (Gd)—for enhanced imaging and targeted treatment.

#### 2.3.1. Gold Nanoparticles (GNPs)

**Figure 5:**
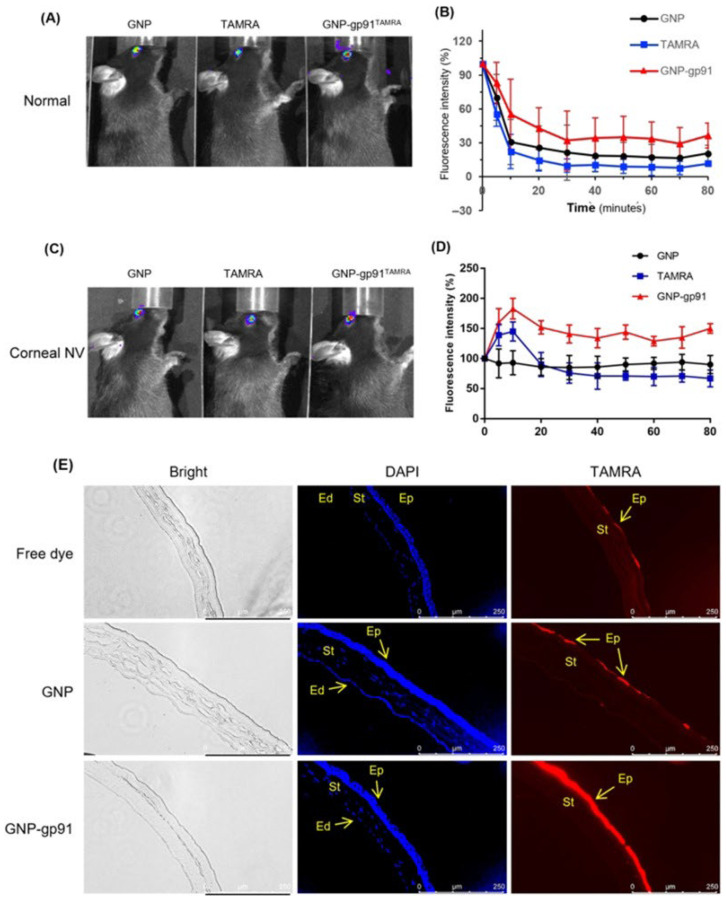
Mechanism of GNPs. Ocular retention and distribution of GNP–gp91. (**A**) Fluorescence images of normal mouse eyes 10 min after treatment with GNP–TAMRA, free TAMRA dye, and GNP–gp91–TAMRA. (**B**) Time-dependent fluorescence intensity changes. (**C**) Fluorescence images of neovascularized mouse eyes 10 min post-treatment. (**D**) Intensity–time profiles. Data represent means ± SD (*n* = 3). (**E**) Cryosections of corneas showing the distribution of free TAMRA, GNP–TAMRA, and GNP–gp91–TAMRA. Yellow arrows indicate dye or nanoparticle locations. Reproduced from Reference [86], under Creative Commons Attribution–Non Commercial (CC BY-NC 3.0 Unported) License. GNPs: gold nanoparticles; gp91: gp91-phox (a subunit of NADPH oxidase); TAMRA: tetramethylrhodamine; GNP-TAMRA: gold nanoparticle–tetramethylrhodamine; SD: standard deviation; CC BY-NC: Creative Commons Attribution–Non Commercial.

GNPs are synthesized through the reduction of HAuCl_4_, with their size ranging from 1 to 120 nm depending on synthesis conditions. Mechanistically, the electron oscillation of GNPs causes local temperature alteration when emitted by laser. As a result, the optical path length of tissues is changed and can be detected by OCT. The sub-micron size of GNPs reduces ocular irritation and scratching while increasing drug corneal permeability [85]. Corneal neovascularization (NV) disturbs the light passing through formerly transparent cornea because of localized abnormal angiogenesis [86,87]. Gp91ds-tat peptide (gp91) competitively binds to p47phox, a regulatory subunit of NADH oxidase, thereby preventing the assembly and activation of NADH oxidase [88]. This peptide inhibits oxidative stress, particularly the Nox2 subtype, thereby reducing cornea angiogenesis [89,90]. Gp91 has a positive charge because it contains lysine and arginine. As a result, type B gelatin with negative charge is used to make gelatin nanoparticles in NV therapy, ensuring close attachment between gp91 and gelatin [86]. Moreover, GNPs can bind to the fluorescent dye TAMRA-SE to measure the retention time of GNP–gp91 on the ocular surface and localize it precisely [86] (Figure 5).

#### 2.3.2. Gd-Based Tracers

**Figure 6:**
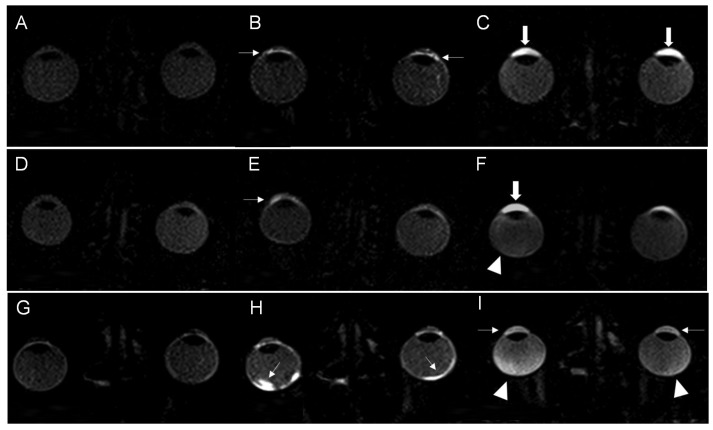
Application of Gd-based tracers. Top row: contrast agent kinetics after Gd injection in healthy patients. (**A**) Native scan; (**B**) 20 min after Gd injection, physiological permeability in the lateral eye chamber; (**C**) 120 min after Gd injection, physiological, symmetric enhancement in the central eye chamber and the VB. Middle row: contrast agent kinetics after Gd injection in right AEC disorder. (**D**) Native scan; (**E**) 20 min after Gd injection, increased Gd enhancement in the central eye chamber; (**F**) 120 min after Gd injection, VB in the right eye. Bottom row: contrast agent kinetics after Gd injection in retinal disorder. (**G**) Native scan; (**H**) 20 min after Gd injection, pathological permeability at the blood–retinal barrier; (**I**) 120 min after Gd injection, accumulation in the VB. Reproduced from Reference [93], Copyright © 2025 by American Society of Neuroradiology. Used with permission. Gd: gadolinium; AEC: anterior eye chamber; VB: vitreous body.

The distribution of Gd-based tracers is visualized through delayed T2-weighted Gd imaging [91]. As a passive permeability tracer, Gd cannot penetrate the blood–aqueous barrier (BAB) normally [92]. Therefore, the abnormal enhancement within the eye on MRI reflects the functional integrity of tight junctions and increased permeability of the barrier pathologically. For example, AEC (anterior eye chamber) diseases, including glaucoma, episcleritis, and uveitis anterior, lead to the higher passage of Gd from anterior to posterior. As a result, about 20 to 120 min after injection of the fluorescent dye TAMRA-SE and gadoteric acid, a stronger signal in the vitreous body (VB), which occupies about four-fifths of the eye’s posterior segment, is shown [93] (Figure 6). PEC (posterior eye chamber) disorders destruct blood–retinal barrier (BRB), making Gd leak from retinal blood vessels. In contrast to AEC disorders, the higher passage of tracers going from posterior to anterior shows a stronger signal in delayed imaging. The advantages and disadvantages of different tracing techniques are listed in Table 2.

## 3. Applications in Ocular Diagnostics

### 3.1. Ocular Tumors

Lymphoma cells exhibit high glycolytic activity, making ^18^F-FDG PET/CT the gold standard for systemic staging in ocular adnexal lymphoma (OAL). It plays a significant role in distinguishing between isolated OAL and systemic lymphoma [70]. Quantitative assessment of tissue radioactivity guides precision medicine and evaluates therapy response. Radiotherapy serves as the first-line treatment for isolated OAL [121], whereas systemic lymphoma requires a comprehensive approach combining surgery, radiotherapy, and chemotherapy [122]. However, physiological ^18^F-FDG uptake by normal periocular structures, including extraocular muscles, lacrimal gland, and lens, obscure the tumor’s signal, complicating the primary diagnosis of intraocular tumors such as uveal melanoma (UM) and retinoblastoma (RB). Research has found that ^18^F-FDG is less sensitive than novel tracers such as ^18^F-AlF-NOTA-PRGD2 in detecting small tumors, resulting from the low metabolic degree of UM [123]. ^18^F-FDG PET/CT is valuable in detecting distant metastasis and optic nerve invasion. For example, ^18^F-FDG successfully identified a rare case of hepatic and osseous metastatic retinoblastoma that might have been missed by conventional imaging [124]. Whole-body metabolic tumor burden in UM can be predicted by quantifying total metabolic tumor volume and total lesion glycolysis on [^18^F]FDG PET/CT [125]. PET/CT allows a whole-body survey in a single scan, offering greater efficiency than performing multiple separate exams such as bone scans, liver MRI, and chest CT. This advantage aligns perfectly with the characteristic of systematic diseases like metastatic tumors that can spread to various sites, including bones, bone marrow, liver, and lymph nodes, and systemic inflammatory diseases. Similarly to tumors, active immune cells rapidly consume glucose to satisfy the great energy need during inflammation [126]. ^18^F-FDG PET/CT is sensitive to metabolically active lymph nodes in endotoxin-induced uveitis (EIU) and sarcoid uveitis, serving as an efficient screening tool [71,110].

N-isopropyl-p-[^123^I] iodoamphetamine (^123^I-IMP) is a γ-emitting radiopharmaceutical used in the diagnosis of atypically presenting uveal melanoma [127,128]. In a cohort of 19 patients, ^123^I-IMP SPECT imaging performed 24 h post-injection achieved 100% diagnostic accuracy for uveal melanoma. All 12 histologically confirmed cases exhibited increased radionuclide uptake. In a cohort of 99 patients with suspected uveal melanoma, ^123^I-IMP SPECT achieved a positive predictive value of 96.3% and a negative predictive value of 97.2% in untreated patients, proving useful for atypical clinical manifestations [127]. Specifically, ^123^I-IMP has demonstrated higher sensitivity than ^18^F-FDG in diagnosing uveal melanoma [129].

A highly sensitive MRI technique is applied to predict optic nerve (ON) infiltration in RB [120]. A 4 cm orbital surface coil improves CNR and spatial resolution compared to a universal head coil, visualizing the small signal increase in TW1. Mechanistically, RB blocks the normal drainage of the orbital glymphatic system, leading to the accumulation of toxic metabolites in the retina. This disrupts the normal microenvironment of the retina and stimulates the release of VEGF, thereby leading to neovascularization. These fragile and highly permeable vessels allow the leakage of Gd to the AC, manifesting as signal enhancement on MRI. ON infiltration is associated with higher risk of intracranial metastasis. Therefore, preoperative MRI can be used in the noninvasive assessment of ON involvement.

### 3.2. Retinal Vascular Disease Evaluation

FA is widely applied in the diagnosis of retinal vessel diseases such as diabetic retinopathy (DR) and enables the visualization of microaneurysms, leakage sites, and non-perfused areas. In diseases such as wet age-related macular degeneration (AMD) with choroidal neovascularization (CNV), fluorescein sodium leaks through the fenestrations of choroidal capillaries. However, as CNV lesions break through the RPE and enter the subretinal space, early abnormal high fluorescence signals and late-stage dye leakage are observed in fluorescein angiography (FFA) images. In choroidal tumors, FFA reveals vascular leakage, vessel occlusion, and the masking effect caused by tumor compression or secondary vascular changes, thereby providing diagnostic clues indirectly [94,95]. When combined with an infrared camera, ICG angiography can be used for choroidal circulation imaging [130]. Therefore, the technique is considered the gold standard for diagnosing and classifying various choroidal vasculopathies [96,97,131]. For example, it can better reveal choroidal occult neovascular membranes than FA and assess vascular choroidal tumors [132].

In white spot syndromes (WSS), FA and ICGA provide distinct angiographic profiles for different subtypes. For example, punctate inner choroidopathy/idiopathic multifocal choroiditis (PIC/iMFC) lesions exhibit early-phase hypofluorescence in ICGA, whereas multiple evanescent white dot syndrome (MEWDS) lesions demonstrate mid- to late-phase hypofluorescence, suggesting pathological involvement at the photoreceptor–RPE interface [37]. Precise lesion localization guides personalized treatment strategies, thereby improving visual prognosis. WF-ICGA has demonstrated high clinical value in CSC. In clinical research involving 151 patients with CSC, it detected asymmetric choroidal venous patterns in 76.8% of cases [133], which helped to predict poor prognoses for patients.

A fluorescent tracer was utilized to achieve dynamic visualization of the distribution of anti-VEGF drugs in neovascular areas in patients through intravenous injection of the near-infrared fluorescent dye 800CW-labeled bevacizumab, providing direct molecular imaging evidence for the evaluation of targeted therapies in AMD [134].

### 3.3. Corneal and Tear Film Dynamics

The core pathological process of glaucoma involves impaired aqueous humor outflow [135,136]. Consequently, a deeper understanding of aqueous humor outflow is essential for developing new drugs that target alternative drainage pathways, thereby reducing intraocular pressure [135]. Gd^3+^ exhibits strong paramagnetism, which significantly shortens the T1 relaxation time of surrounding water protons, leading to the enhancement signal on T1-weighted MRI. They diffuse and follow aqueous humor, which ultimately accumulates in the posterior chamber. The mechanism is used to measure aqueous humor dynamics based on data such as peak enhancement and rate of initial rise, contributing to quantitative evaluation of aqueous humor dynamics under various antiglaucoma therapy. Timolol maleate is a frequently used IOP-reduced medicine glaucoma therapy. A nonselective β-blocker, timolol inhibits the generation of aqueous humor, thereby limiting the availability of Gd in the anterior chamber. This alteration is reflected in the Gd signal curve via the lower initial rate of signal increase and reduced area under the curve. Serving as a selective β-blocker, brimonidine not only inhibits the generation of aqueous humor, but also promotes uveoscleral outflow [137]. Gd-MRI has shown higher peak Gd intensity in both eyes, indicating that brimonidine destroys the integrity of the aqueous–vitreous barrier and blood–ocular barrier [138]. This finding might explain the systemic side effects, such as CNS depression, hypotension, and bradycardia, resulting from systemic absorption of topically applied drugs. Stable isotope-labeled water enables non-invasive and quantitative tracking of intraocular fluid dynamics. After intravenous injection of deuterated water (D_2_O), the use of high-sensitivity MRI to continuously monitor its accumulation and clearance processes in the anterior chamber and vitreous body allows precise analysis of the permeability characteristics of the blood–aqueous barrier, the rate of aqueous humor production, and fluid exchange differences between different ocular compartments [139]. It is helpful for in vivo functional imaging for investigating the mechanisms and evaluating the therapeutic effects of eye diseases associated with abnormal fluid metabolism, such as glaucoma and retinal edema.

**Figure 7:**
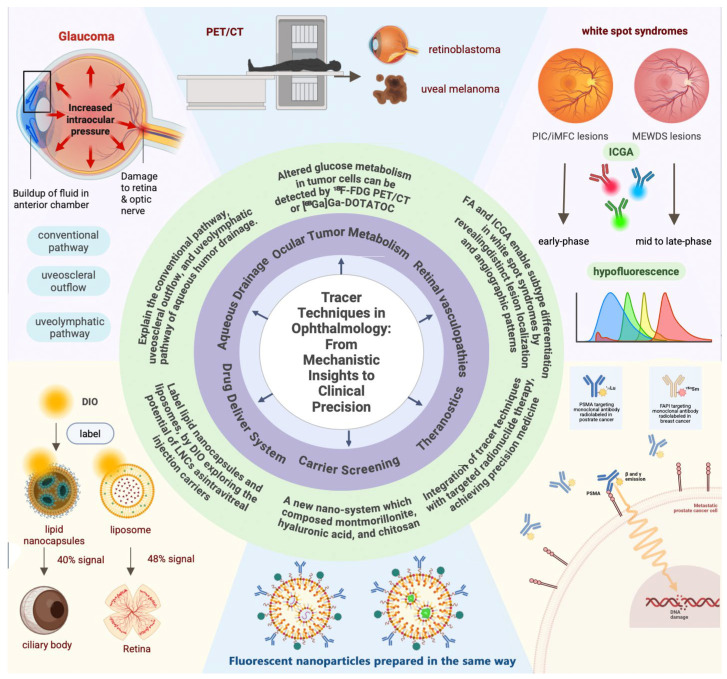
Tracer techniques in ophthalmology: from mechanistic insights to clinical precision PET/CT, positron-emission tomography/computed tomography; FDG, fluorodeoxyglucose; PIC, punctate inner choroidopathy; MC, multifocal choroiditis; MEWDS, multiple evanescent white dot syndrome; ICGA, indocyanine green angiography; FA, fluorescein angiography; PSMA, prostate-specific membrane antigen; FAPI, fibroblast activation protein inhibitor; LNC, lipid nanocarrier.

The clinical application of tracer techniques in ophthalmology is illustrated in Figure 7.

## 4. Applications in Systemic Correlation

### 4.1. Eye–Brain Axis

#### 4.1.1. Neural Tracers: Anterograde, Retrograde, and Transsynaptic Tracers

The eye is closely associated with the brain. As part of the CNS, the eye has the same embryonic origin as the brain, and the three layers of meninges that wrap it are a direct extension of the brain. Besides anatomical connection, visual information is transmitted to multiple target regions in the brain, such as the superior colliculus and lateral geniculate nucleus. Pathological changes, including amyloid-beta (Aβ) deposition, tau tangles, neuroinflammation, RGC loss, and vasculopathy, occur synchronously in the retina, and their severity correlates with brain pathology and cognitive decline. The eye–brain axis is helpful in understanding visual processing, restoration, and mechanisms of ocular disorders and neurodegenerative diseases, thereby achieving the early screening, early diagnosis, and early treatment of neurodegenerative and ocular diseases.

In 1971, research on innate transport systems of cells utilized tracers within neurons, thus marking the axons’ origin, path, and termination points. According to the transport direction and transsynaptic capability, we divide the neural tracers into three categories: anterograde, retrograde, and transsynaptic.

**Figure 8:**
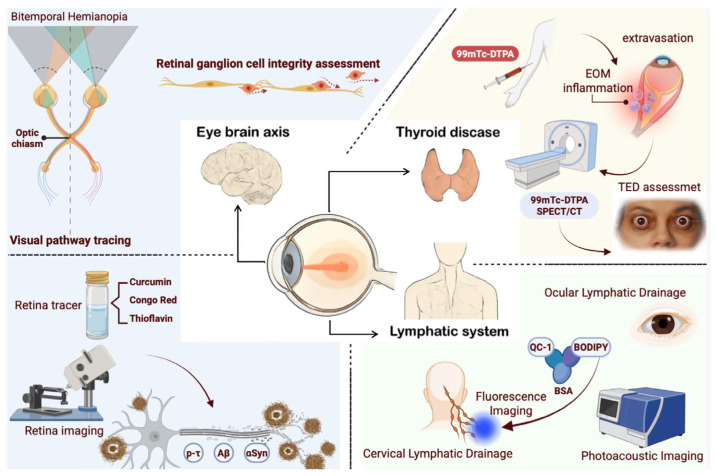
Tracers connect eye and systematic diseases. ^99^mTc-DTPA, technetium 99m diethylenetriaminepentaacetic acid. TED, thyroid eye disease. EOM, extraocular muscle. BODIPY, boron–dipyrromethene. QC-1, quinazoline chemical 1. BSA, bovine serum albumin. SPECT/CT: single-photon-emission computed tomography/computed tomography.

Taken up by neurons at the injection site, anterograde tracers are transported from the cell body toward the axon terminals. [^3^H] leucine or [^3^H] proline are the first tracers applied in anterograde tracing. Radioactive tracers were injected into the vitreous humor of monkeys, and were taken up by RGCs. After autoradiography of brain sections, a strong and dense signal was found in the caudal superior colliculus (SC), indicating that the retinocollicular projection was weighted strongly to the peripheral visual field [140]. Retrograde neural tracers selectively transduce functional genes, such as ChR2 [141], GCaMP [142], and DREADDs [142], into projection neurons, achieving the visualization and modulation of neuronal networks. The tracing process involves the uptake of tracers by nerve terminals and then centripetal transport in first-order neurons. The natural serotype AAV11 has been shown to have a higher astrocyte target efficiency in dHPC than the conventional serotype AAV5 [143]. The dysfunction of astrocytes includes inflammatory responses and abnormal glutamate uptake, driving the onset of neurodegenerative diseases such as AD and Parkinson’s disease. AAV11 can serve as a vector in astrocyte-targeted therapy in neurodegenerative disease. FluoroGold has been used to label and count RGCs in the retina under intense light exposure [144]. The dye was injected into the SC of rats, taken up by the axon terminals of RGCs, and retrogradely transported to their cell bodies. Finally, the number of tracer-labeled RGCs was visualized in flat-mounted retinas. FluoroGold also serves as an assessment tool for the survival and integrity of RPEs and retinal neurons [145]. This is illustrated in Figure 8.

Transsynaptic tracers are transported to the neuron bodies and start to infect after being taken up by nerve terminals. Neurotropic viruses are frequently used in transsynaptic tracers, which produce controlled infections. The replication of the virus then kills the infected neurons or becomes a latent infection state. Finally, the bud of the virus is transported to nearby neurons through synapses. Adenoassociated virus (AAV) serotype 1 encoding a Cre recombinase and H129-HSV are two widely used neuron traces [146,147]. However, they may exhibit disadvantages such as nonspecific retrograde tracing, high toxicity, limited capacity, and weak signal [148]. For example, the inadequate labeling intensity of H129dTK-TT and H129-EGFP failed to visualize overall neuronal morphology and fine details. Novel fusion tracers are increasingly being used in eye–brain axis research, addressing these limitations. H129Amp is a pseudoviral tracer system derived from the HSV-1 H129 strain, which is composed of the H129Amp particle and helper virus. The H129Amp particle contains Cre recombinase, the HSV-TK gene, which is required for replication of the helper virus, and green fluorescent protein, which labels neurons. Because H129Amp lacks viral replication genes, a helper virus is equipped with the full set of viral proteins enabling replication and packaging of the tracer genomes inside starter neurons, which can enhance signal strength and guarantee monosynaptic specificity. Another example is the genetically encoded neuron tracer mWGA-mCherry (mWmC). Adenoassociated virus (AAV) serves as a gene-delivery vehicle that specifically infects RGCs and expresses the mWGA-mCherry fusion protein [149]. mWmC shows minimal retrograde spread because of its unique structure of C-terminal fusion, which tucks mCherry intracellularly. The pKa of mCherry is low (~4.5), making it fluorescent in acidic lysosomes and tolerant of lysosomal proteases. As a result, mWmC exhibits bright perisomatic puncta after endocytosing into lysosomes in transsynaptic transfer. This characteristic solves the dimmer labeling of conventional tracers such as WGA and tdTomato with higher pKa. mCherry is also used in proving monosynaptic connections from GABAergic RGCs to parvalbumin-positive (PV^+^) neurons in SC as fluorescein [150]. mCherry is stably expressed during replication, enabling the reproducibility of tracing results.

In neuro-ophthalmology, it is important to evaluate ON injury levels and intervene in timely fashion because of the irreversibility of RGCs. Numerous neuroprotection agents, such as neurotrophins, memantine, nicotinamide, and growth hormone (GH), have been reported to intervene in the neuronal death pathway, thereby preventing RGC damage in glaucoma [151]. Transsynaptic anterograde tracing can assess neural function, including integrity and regenerative potential. CTB-AF488, the conjugate compound of cholera toxin subunit B and a dye, has been applied to investigate the neuroprotective effects of GH following ON crush injury in rats [152]. CTB specifically binds to GM1 ganglioside receptor and then is taken up actively into neuron bodies by RGCs. The transport of CTB is an energy-dependent process. Therefore, detecting CTB in ON demonstrates the normal metabolic and transport functions of the axons. The dye AF488 emits bright green fluorescence when excited by specific wavelength. After intravitreally injecting CTB-AF488, the surviving RGCs actively uptake and transport the tracer anterogradely towards the brain. Therefore, the CTB fluorescence signal observed in ON reflects the number of functional axons. A transsynaptic tracer is not only a labeling tool, but also a functional probe capable of evaluating the integrity of neural pathways and efficiency of neuroprotection agents.

#### 4.1.2. Retinal Molecular Biomarkers for AD

Research has found that Alzheimer’s disease (AD) patients often have early visual impairments simultaneously [153], resulting from pathological alterations including retinal blood vessel narrowing, glial cell activation, and retinal ganglion cell degeneration [154,155].

Retina tracers’ emergence as promising early sites for AD pathology enable noninvasive detection and early intervention—even before clinical symptoms. Aβ accumulation in the retina of AD patients has been reported, which causes neurodegeneration in the retinal ganglion cell layer. Plaques in the eye and brain originate from the abnormal breakdown of amyloid precursor proteins. Aβ plaques autofluoresce bright blue when excited by 360- to 370-nanometer UV light, allowing for the direct detection of Aβ without exogenous labels [156]. This feature is promising in autofluorescence tracing in early AD diagnosis, reducing tissue damage upon tracer injection. A human Aβ (hAβ) tracer is used to investigate abnormal Aβ accumulation. It has been found that this accumulation results from centrifugal transport of Aβ from the brain to the eye [157]. Exogenous and fluorescently labeled hAβ was injected into CSF through the cisterna magna (CM). Then, hAβ was transported along the ON via the subarachnoid space (SAS) and subsequently entered the ON, where it advanced through both the perivascular space (PVS) and the axonal compartments toward the retina. This pathway reminds us that the eye is serving as the window of the brain and holds significant potential for presymptomatic diagnosis.

Curcumin is another frequently used molecular tool applied in retinal imaging for AD [158]. A natural fluorophore, curcumin crosses through BRB and binds to Aβ fibrils and oligomers specifically in the retina. Then, the complexes are detected by confocal scanning laser ophthalmoscopy (cSLO) equipped with a blue excitation laser that optimally excites the curcumin fluorophore. It has been reported that there are an increased number of fluorescent spots in AD [155]. The retinal amyloid index (RAI) is a quantitative metric calculated by the number, size, and fluorescence intensity of fluorescent spots, achieving the measurement of structural alterations such as hippocampal atrophy.

In vitro tracing offers nanometer-scale resolution, which can be used to validate the results of in vivo tracing [159]. This process contains the immersion-fixed, dehydrated, and paraffin-embedded tissue for histology to confirm that the fluorescent signals detected correspond to pathological tissues. Common in vitro tracers include thioflavin, Congo red (CR), Gallyas silver stain, and curcumin [98] (as illustrated in Figure 8). Thioflavin T (ThT) is regarded the gold-standard method to investigate aggregation and misfolding of proteins underlying the mechanism of neurodegenerative processes including AD and Parkinson diseases (PD) [160]. The fluorescence density of ThT is increased after it binds to β-sheet-rich structures. Mechanistically, in low-viscosity environments, ThT rotates rapidly around the carbon–carbon bonds. As a result, the energy is almost entirely dissipated into the surrounding solvent through molecular collisions, leading to nearly indetectable fluorescence. After the binding, the rotations are greatly restricted, resulting in an intense fluorescence signal. CR also can intercalate into cross-β-sheet structures, which exhibit apple-green birefringence under polarized light [155]. Another example is ThS, which can be transported through inhalation in Aβ detection [161]. Besides neurodegenerative diseases, these tracers can also be applied to the detection of amyloid-like aggregation in cataractous lenses [162]. Detailed information on eye–brain axis tracers is shown in Table 3.

**Table 3:** Eye–brain axis tracers.

Tracer Type		Field of Application	Principle	Clinical/Research Application Example
**Neural tracer**	Anterograde Tracer	Eye–brain axis research	Taken up by neurons at injection site Transported from soma to axon terminals Accumulates in axon terminals	Monitoring rat neural circuit (auditory cortex to medial geniculate nucleus and lateral amygdala) [163] Investigating monkey retinocollicular projection with [^3^H] leucine/proline [140] Detecting functional axon numbers with CTB-AF488 [164]
	Retrograde Tracer	Eye–brain axis research Neuroscience	**Uptake** at the target site through receptor mediated endocytosis: Binding Pit formation Vesicle scission Uncoating **Retrograde transport** via microtubules (dynein-mediated) **Accumulation** in the somata of first-order neurons	Mapping neural circuits in AD models with H129-dgK-G4 [165] Simultaneous multi-pathway tracing with NNTs [166] Labeling cellular structures (microtubules, lysosomes) with DBDNC-NPs [167]
	Transsynaptic Tracer	Eye–brain axis research Neural connectivity map	**Rabies virus:** Binding between VSV-G protein and PS Cell membrane invagination and endosome formation pH drop induces conformational change in G protein Membrane fusion and viral genetic material release Retrograde transport **AAV or H129:** Binding between gD protein and receptors (Nectin-1 or HVEM) Internalization Replication of viral genomes Assembly Anterograde Transport Secondary infection	Studying monosynaptic connectivity (GABAergic RGCs to PV^+^ neurons in SC) with mCherry [150] Tracing mouse mPFC to striatum with ATLAS [168] Transsynaptic labeling (retina to visual centers) with AAV1-Cre [147] Labeling higher-order neurons (olfactory/visual systems) [146] Investigation smell-control stress responses [169] Investigation of expression of local taste receptor genes in gustatory ganglia and brain [170]
**Retinal tracer**	hAβ tracer	Early diagnosis of AD Ocular disease research	Core structures (benzothiazole, quinoline, benzimidazole) intercalate between β-sheets of Aβ plaque Formation of π–π stacking	Simulation pathological process of late-onset sporadic AD [171] Detection of retinal plaque deposit in early AD [157] Prediction of visual impairment [157]
	Curcumin	Early diagnosis of AD	Insertion of β-diketone into β-sheet cavity π-π stacking Blue-light excitation and green fluorescence emission Antioxidant and anti-inflammatory properties	Detection of retinal plaque deposit in early AD [155] Investigating therapy for autoimmune uveitis [172] Neuroprotection of RGCs in glaucoma [173] Decreasing ROS and TNF-α of RPE cells for DR [174]
	ThT	Neuroscience	Rapid rotation around carbon–carbon bond in free state Energy dissipation through molecular collisions Restriction of rotations upon binding to aggregates Intense fluorescence emission after binding	Monitoring kinetics of fibril formation of α-synuclein [175] Gold-standard method for aggregation processes [160]
	ThS	Early diagnosis of AD Ocular disease research	Similar to ThT, but with higher Brightness Affinity Lipophilicity	Aggregation processes [176] Detection of retinal plaque deposit in early AD [176]
	CR	Neuroscience Cataract research	Binding to cross-β-sheet structure of amyloid fibrils Exhibits apple-green birefringence under polarized light	Detection of retinal plaque deposit in early AD [177]

AAV: adenoassociated virus. AD: Alzheimer’s disease. CR: Congo red. CTB-AF488: cholera toxin B subunit conjugated with Alexa Fluor 488. DBDNC-NPs: donor–bridge–acceptor-based near-infrared convertible nanoparticles. DR: diabetic retinopathy. gD: glycoprotein D. hAβ: human amyloid-β. HVEM: herpesvirus entry mediator. mPFC: medial prefrontal cortex. NNTs: nanoneedle tracers. PV^+^: parvalbumin-positive. RGCs: retinal ganglion cells. ROS: reactive oxygen species. SC: superior colliculus. ThS: thioflavin S. ThT: thioflavin T. TNF-α: tumor necrosis factor α. VSV-G: vesicular stomatitis virus glycoprotein.

### 4.2. Thyroid Disease

Thyroid eye disease (TED) is a systematic autoimmune disease affecting orbital and periorbital tissues. The course of disease is divided into an active phase, which is characterized by inflammation infiltration, and an inactive phase, which is characterized by chronic fibrosis [178]. Multiple radionuclides are applied in the assessment of inflammatory activity in TED [179]. After the intravenous injection of ^99^mTc-DTPA, it extravasates at the inflammation site in extraocular muscles and then binds specifically to polypeptides in inflammatory fluid. The integration technique ^99^mTc-DTPA SPECT/CT serves as a tool in lacrimal gland and extraocular muscle inflammation assessment in TED [78]. An example is shown in Figure 8. Ga is another new tracing technique in TED assessment. [^68^Ga]Ga-DOTATOC and [^68^Ga]Ga-DOTATATE exhibit high affinity for somatostatin receptors (SSTRs) [180]. Somatostatin (SST) is a cyclic polypeptide hormone widely present in the human body that inhibits the secretion of various other hormones and cell proliferation. SSTRs are upregulated in activated lymphocytes, orbital fibroblasts, and meningioma cells under pathological conditions characterized by hyperfunction, such as elevated metabolism, hormone secretion, or cellular proliferation. Therefore, radioactive tracers can also be applied in the assessment of TED inflammatory activity [180,181].

### 4.3. Lymphatic System

The eye is suitable for lymphatic system research because its location is superficial and it has complex drainage pathways, such as the parotid lymph nodes, submandibular lymph nodes, and deep cervical lymph nodes. Therefore, injecting tracers under the conjunctiva or onto the ocular surface achieves direct visualization of the lymphatic drainage pathway. This method is simple and causes minimal damage [182]. Moreover, the drainage pathway of aqueous humor is closely related to the lymphatic system. Proteins and particles in aqueous humor can be cleared via the prelymphatic pathway. Multiple tracers are applied to study drainage pathways, including conventional pathways, uveoscleral outflow, and the uveolymphatic pathway.

In a PAI study investigating the controversial uveolymphatic pathway, a photoacoustic tracer was formulated using QC-1 as the dye and bovine serum albumin (BSA) as the carrier protein to enhance solubility and biocompatibility. The specific molecular weight of QC-1/BSA facilitates its preferential uptake by lymphatic system rather than capillaries. BSA has become a widely used carrier in exploring ocular lymphatic drainage. Another study utilized QC-1/BSA/BODIPY (QBB), a complex synthesized by conjugating QC-1 and BSA and subsequently reacting it with BODIPY FL maleimide, for quantitative imaging of ocular lymphatic drainage [106]. In addition to in vivo PAI demonstrating the patency and function of the lymphatic pathway from the eye to the right cervical lymph nodes, ex vivo fluorescence imaging was performed to detect BODIPY-derived signals [183]. The hybrid tracer enabled dual-modality imaging, combing quantitative capability in deep tissue of PAI with high-resolution anatomical validation of fluorescence imaging [184,185]. Promisingly, it can be applied to lymphatic drainage research in cancer tissues, including sentinel lymph node biopsy and tumor metastasis tracing [186].

In vivo tracing studies have confirmed that the eye is not an immune-privileged island. Moreover, aqueous humor and cerebrospinal fluid (CSF) from the optic nerve sheath converge along the Virchow–Robin spaces and drain into the deep cervical lymph nodes [187], forming the “eye–brain–neck” axis. The injection of optical tracers, including QBB, QC-1/BSA, and fluorescently labeled dextran, into the vitreous chamber (IVT) or anterior chamber (AC) provides anatomical validation of this pathway [188]. This example is illustrated in Figure 8. Furthermore, dysfunction of ocular lymphatic drainage, which serves as an exit route for the brain’s glymphatic system, can lead to the accumulation of aberrant proteins, including tau aggregates, β-amyloid, and α-synuclein, which is directly linked to the pathogenesis of neurodegenerative diseases such as Alzheimer’s, Parkinson’s, and glaucoma [189,190,191].

## 5. Challenges and Limitations

### 5.1. Off-Target Binding

Off-target binding refers to the non-specific interaction of a tracer with unintended cell types or molecules, compromising diagnostic accuracy and potentially leading to adverse events [192]. For example, neural tracers such as CTB can effectively track RGCs, but also label amacrine cells, because GM1 exists in both RGCs and amacrine cells non-specifically. As a result, the RGC density is overestimated, which leads to less accurate calculations of RGC loss rates in disease monitoring. Specifically, CTB labeling overestimated RGC density by 19.4% compared to the RGC-specific marker RBPMS. To mitigate this off-target effect, co-staining with RGC-specific markers, including RBPMS and Brn3a, is recommended. Only 53–58% of CTB^+^ cells co-localized with RBPMS, indicating significant off-target labeling. Co-staining enables subtraction of false-positive signals and allows correction factors to be applied for more accurate RGC quantification [193].

Off-target binding has the potential to cause unintended cellular toxicity, adverse events, and clinical trial failures [194]. The similar challenge also exists in retinal tau and amyloid imaging. The detected signal density relies on the changes in the retinal cellular microenvironment including metabolic dysregulation, oxidative stress, and pathological protein crowding. However, numerous retinal disorders, including AMD and diabetic retinopathy, share the pathological processes of oxidative stress, leading to damage to proteins, lipids, and DNA from reactive oxygen species (ROS). As a result, the altered overall fluorescence signal might not distinctly link to AMD [98].

Similar circumstances exist in AD diagnosis because retinal Aβ accumulation is not specific. AMD and glaucoma also involve progressive accumulation of Aβ plaques and hyperphosphorylated tau protein, restricting the specificity of Aβ serving as the early screening tool for presymptomatic AD [131]. Therefore, we recommend the use of multi-biomarker panels for differential diagnosis. For instance, combining Aβ detection with p-tau, neurofilament light chain (NfL), or inflammatory markers would significantly improve diagnostic specificity. Research shows that single biomarkers show substantial test-retest variability, ranging from 4.1% for Aβ42/Aβ40 to 25% for GFAP. In contrast, multi-biomarker combinations achieve higher diagnostic accuracy and reduce the proportion of unstable predicted outcomes to only 14% [195].

### 5.2. Safety Concerns

Safety concerns present another challenge during the translation of experimental tracing techniques into clinical applications, which is restrictive for patients requiring repeated examinations within a short period of time or multi-tracer imaging protocols.

#### 5.2.1. Radiation Dose Toxicity

Many chronic progressive eye diseases, such as DR, uveitis, and intraocular tumors, require long-term and repeated imaging surveillance to assess disease progression. However, the cumulative radiation dose associated with radioactive tracers limits their frequency when used as the primary monitoring tool. Among patients undergoing serial ^18^F-FDG PET/CT scans, the median effective dose per examination was 25.1 mSv, and 12–13% of patients received a cumulative dose ≥100 mSv, with the 25th percentile of two-year cumulative dose reaching 109 mSv. For patients with DR or uveitis requiring biannual surveillance over 10 years, the cumulative dose would approximate 100–200 mSv, corresponding to a lifetime attributable cancer risk of 0.5–1.0% [196].

It may also increase the risk of leukemia [197] and radiation-induced cataracts [198]. This compels clinicians to make difficult trade-offs between the diagnostic benefits gained and the potential radiation hazards involved. Xerostomiais is the most commonly reported side effect of PSMA-targeted radioligand therapy, impairing patients’ life quality. The incidence of dry mouth was observed to be 39% in the phase III trial of [^177^Lu] Lu-PSMA-617 [199]. In PSMA-targeted alpha therapy, the incidence of Xerostomiais is nearly 100% [200]. Mechanistically, glutamate carboxypeptidase III, whose structures are similar to PSMA, is expressed in salivary glands and leads to the accumulation of radiopharmaceutical [201].

The ocular and orbital tissues in children are in a phase of rapid development, with highly active cell division, making them far more sensitive to radiation than adults. Research has shown that effective doses in children range from 6.15 to 15.28 mSv, with a five-fold higher lifetime cancer risk per unit dose than adults [202].As a result, even low-dose radiation exposure may exert unpredictable effects on the development of the lens, retina, and optic nerve, while also significantly increasing the risk of tumor development later in life. As a result, the use of radiopharmaceuticals in pediatric eye diseases—such as for evaluating the efficacy of retinoblastoma treatment or monitoring congenital intraocular inflammation—is approached with extreme caution.

#### 5.2.2. Invasive Injection Damage and Chemical Toxicity

Conventional fluorescent tracers have prolonged tissue retention. For instance, ICG persists in fundus after vitrectomy and is cleared slowly in diabetic retina [203]. Optical coherence tomography angiography (OCTA) is a non-invasive imaging modality that visualizes retinal and choroidal vessels without dye injection or radiation exposure. It can penetrate the retinal layer and provide a 3D image of various plexus of capillaries, providing evidence for diagnosis of DR, diabetic macular edema, and retinal vein occlusion [204]. Unlike fluorescent tracers or radionuclides, OCTA eliminates risks of chemical toxicity, radiation exposure, and injection-related complications, making it particularly suitable for patients requiring frequent monitoring, such as those with DR or uveitis.

Though invasive injection, such as intravitreal injections, increases bioavailability greatly, the infection, injection pain, and uneven drug distribution limit its widespread adoption [205]. The injection of fluorescent dyes might lead to side effects such as nausea, vomiting, allergy and urine discoloration [206]. The retina and lens are extremely delicate and highly sensitive to toxic substances. Tracers such as synthetic fluorescent dyes, quantum dots, or radionuclide exist inherent cytotoxicity or phototoxicity. For instance, tracers induce inflammatory responses, oxidative stress, and even lead to apoptosis of RPE cells under prolonged exposure, potentially accelerating the progression of AMD.

### 5.3. Blood–Ocular Barrier Penetration Variability

The eye has complex physiological barriers, such as the BRB and BAB, restrict the passage of macromolecules, toxins, and inflammatory mediators, ensuring highly selective permeability. However, it also restricts administration route of tracers. Topical administration is easily washed away less than 15–30 s after instillation because tear volume restores rapidly, with repeated blinks resulting from corneal stimulation.

### 5.4. Instability to Interference in Imaging

In ophthalmic applications, fluorescence imaging exhibits artifacts due to optical media opacities, such as cataracts, vitreous opacities, or corneal scars. These artifacts primarily manifest as signal attenuation, non-specific scattering, and reduced spatial resolution. Such optical interference can not only obscure genuine pathological signals, such as retinal neovascularization or inflammatory lesion, but may also introduce bias in the quantitative analysis of fluorescence intensity, thereby compromising the accuracy of disease diagnosis and treatment efficacy evaluation [207]. Photobleaching, characterized by rapid fluorescence decay under continuous laser illumination, serves as another obstacle. This requires short signal acquisition times to avoid compromised data and makes long-term dynamic monitoring impossible. These drawbacks have contributed to the development of more advanced tracing techniques [106].

### 5.5. Challenges in Clinical Translation

Despite tracers having been utilized in basic research and preliminary clinical applications, many challenges remain in clinical translation, including ligand selectivity, tissue distribution, biocompatibility, dose optimization, regulatory approval, and scalable manufacturing [208,209]. Ocular tracer design should consider ocular physiological barriers and delicate structures, such as the tear film barrier, the tight junctions of the corneal and conjunctival epithelium, and the blood–ocular and blood–retinal barriers, which is crucial for selecting efficient DDSs [210]. Scalability and manufacturing feasibility are important. Formulation design should anticipate future large-scale production and compliance with good manufacturing practice (GMP) standards, including specific quality control metrics such as radiochemical purity (>95%), specific activity (>10 GBq/μmol for radiotracers), and endotoxin levels (<5 EU/kg) [211], as minor variations during nanomedicine manufacturing significantly impact their physicochemical properties. These properties include particle size, shape, composition, crystallinity, drug loading capacity, and drug release profile [212]. To achieve this goal, research institutions proactively engage with regulatory agencies like the FDA or EMA early on and request pre-investigational new drug (pre-IND) meetings. Such engagements can provide early guidance on tracer design, regulatory requirements, and preclinical data expectations.

Additionally, even for tracers with therapeutic potential, high manufacturing and quality control costs, regulatory approval expenses, and significant industrialization barriers hinder their clinical adoption [213]. Multicenter trials are essential for demonstrating the applicability, safety, and stability of new tracers, which requires collaboration among drug developers, clinicians, pharmacokinetic expert, and drug regulatory authorities [213].

In summary, though basic research and animal model studies show great potential for tracers, advancing to future clinical trials requires not only mature core technology, such as well-established technology of probe design, targeting, and safety, but also overcoming clinical translation issues. These include the standardization of manufacturing processes including GMP implementation, navigation of regulatory and ethics approvals, assessment of cost-effectiveness, and clinical validation through multicenter trials with large samples. Only when these challenges are solved can newly designed tracers be widely applied and provide reliable support for precision medicine in ophthalmology.

## 6. Future Directions

### 6.1. High-Targeting, High-Safety, High-Stability Tracers

In the future, the development of novel ocular tracers that integrate both high biosafety and superior performance is critical and essential.

#### 6.1.1. High Targeting

Higher retention rates in targeted tissues are desirable, yet lower residual retention time of tracers in non-target tissues remains essential. Multiple novel tracers have been developed to achieve a higher retention rate of targeted tissue. Based on this principle, research has developed multiple tracers, including ^99^mTc-ZIGF1R:4551-GGGC probe in Graves’s ophthalmopathy [214], [^64^Cu]Cu-NOTA-ABDB6 in the visualization of CD70-positive lymphoma [215], [^99^mTc]Tc-MY6349 in the therapy of trophoblast cell-surface antigen 2 (Trop2) target in breast cancer [216]. In the future, more ocular tracers with higher retention rates in targeted tissues are required in precise medicine.

#### 6.1.2. High Safety

Ultraminiature chain-like gold nanoparticle clusters (GNCs) represent a class of inorganic nanotracers capable of renal clearance via urine [59]. Unlike conventional nanoparticles, which raise biosafety concerns due to hepatic accumulation, GNCs exhibit minimal organ retention and low cytotoxicity. Therefore, they are promising in tracing choroidal neovascularization and monitoring the transplantation of human induced pluripotent stem cell-derived retinal pigment epithelium (hiPSC-RPE) [59,217]. In AMD patients, novel fluorescent tracers provide an early and sensitive way to localize transplanted hiPSC-RPE and evaluate therapeutic efficacy of steam cell ocular therapy.

In the future, radioactive tracers will advance toward lower doses and higher precision. The core of this trend lies in achieving a significant reduction in radiation exposure for patients without compromising diagnostic information quality. Promisingly, dual-tracer PET/CT imaging strategy has been shown to reduce radiation exposure. For example, 10% of the standard [^18^F] FDG dose is injected first, followed by an 84.6 ± 28.0 min wait before the [^18^F] FDG PET/CT scan. After this scan, [^68^Ga] Ga-FAPI-04 of half-activity is injected immediately, followed by a 60 min dynamic PET scan [218]. This new protocol has been proved to reduce the radiation exposure from both of PET and CT.

While intravenous fluorescein angiography (IVFA) remains the standard for retinal imaging, its invasiveness limits repeated use, particularly in pediatric patients. Oral fluorescein angiography (OFA) using 25 mg/kg of 10% fluorescein provides high quality of diagnosis for various retinal diseases. Only 0.019% mild adverse effects are reported, offering a less invasive alternative to IVFA [219].

#### 6.1.3. High Stability

Novel fluorescent tracers are composed of fluorophores conjugated with targeting structures, including nanobodies, monoclonal antibodies, and small peptides, that enhance safety and superior detection sensitivity [220]. For instance, a hydrogel-based lacrimal plug embedded with ICG nanoparticles has been used in dry eye treatment [99]. This design enables long-term monitoring of occlusion efficacy and duration by measuring the attenuation of the fluorescence signal, which indicates the hydrogel’s degradation degree. This approach mitigates the treatment failure caused by plug displacement [221].

### 6.2. AI-Driven Tracer Techniques Development

The neural network of deep learning (DL) enables imitation of the information processing of human neurons. The process involves the training of machines with numerous images and enables to process and classify information and predict results without human intervention [222]. The combination of DL with tracer techniques achieves the high efficiency screening of early-stage disease [22]. An autonomous AI-based diagnostic system has shown up to 90% in both specificity and sensitivity in DR screening [223]. The comprehensive AI-based screening will greatly save time and money, enable mass screening in the general population, and increase the quality of healthcare while avoiding missed diagnoses and misdiagnoses caused by false negatives and false positives. The property is suitable for the screening of avoidable blindness in DR, AMD, and glaucoma. The practicability of a DL system with fundus images featuring a 45° field of view has been approved in a rural area population [224]. The promotion of AI-driven tracer techniques in rural areas decrease the reliance on ophthalmologists and save time and money for screening because the population is huge and the demand for health care is high, a conclusion drawn from the increasing blindness incidence and prevalence of ocular diseases in Handan and Andhra Pradesh [225,226]. The eye is regarded as the window of the system. The retinal vessels are densely distributed across the fundus, reflecting the characteristics and regulation of the systematic blood vessels [227]. Multiple disease prediction algorithms have been applied to analysis CFPs outcomes, measure parameters such as retinal-vessel caliber, and predict the occurrence of various disease such as dementia [228], cardiovascular diseases [229], and diabetes [230].

### 6.3. Multimodal Integration

Single imaging modalities suffer from interferences such as unstable signal intensity, scattering and artifacts. The integration of multiple imaging modalities provides a comprehensive understanding in biological and medical research. For example, one research study utilized pharmacodynamic to confirm the efficacy of a newly developed NP formulation that was proved to increase the bioavailability of an anti-glaucoma drug using fluorescence tracing [48]. Through continuous monitoring of IP fluctuations, the study found that the pressure-lowering effect over a 12 h period was better than a traditional formulation, which confirms its sustained efficacy. The integration of tracing data with computational modeling contributes to drug development. For example, tracing data were applied to calibrate and validate a quasi-3D (Q3D) model that simulated drug transport across the in vitro rabbit cornea [182]. The model predicted the spatiotemporal distribution of both hydrophilic and lipophilic tracers (fluorescein and rhodamine (B) in the cornea, which were closely related to the three barriers of the cornea: the epithelium, the stroma, and the endothelium. Q3D enables us to predict the penetration ability of candidate drugs, thereby prioritizing molecules which are both hydrophilic and lipophilic and reducing development costs and time.

Multimodal integration has shown potential in the diagnosis and treatment of ophthalmic diseases. Within the pachychoroid disease spectrum, the combination of imaging techniques such as OCT, OCTA, and ICGA provides complementary insights spanning structure to dynamic blood flow and from morphological appearance to functional mechanisms. Specifically, OCT captures choroidal structural abnormalities, ICGA reveals pathological features of vascular permeability, and OCTA provides noninvasive visualization of dynamic microvascular networks [231]. As a result, multimodal integration will greatly facilitate the transition toward precision medicine in clinical management.

### 6.4. Clinical Transition

Clinical translation of tracers remains a critical challenge. In this review, we have summarized the preclinical and clinical applications of common ocular tracers, as well as the barriers encountered during clinical translation (Table 4). To achieve better clinical transition of ocular tracers, a standardized multi-center trial roadmap should be established. Phase I focuses on safety, biodistribution, and ocular tolerance in healthy volunteers. Phase II expands to patients and evaluates different administration routes, including topical, intravitreal, and subconjunctival. Phase III conducts large-sample, randomized, controlled multicenter trials to validate diagnostic accuracy, inter-center consistency, and superiority over conventional imaging [232].

Although tracer-based imaging such as SPECT is expensive (approximately USD 1900 per scan), its cost-effectiveness should be evaluated especially in the management of chronic disease management including DR, UM, retinoblastoma, and neurodegenerative disorders [237]. Early detection with tracers can prevent irreversible damage and reduce long-term socioeconomic burden. For instance, delayed diagnosis of Parkinson’s disease costs an estimated USD 51.9 billion annually in the United States [238]. However, the necessity of tracer screening must be critically assessed when alternative low-cost approaches provide adequate information. Therefore, the necessity of routine tracer use in low-risk populations needs to be evaluated.

Early engagement with regulatory agencies, including the FDA, EMA, and NMPA, through pre-IND meetings, pursuit of orphan drug designation for rare ocular diseases, and harmonized global regulatory frameworks will facilitate clinical translation and accelerate patient access to innovative tracer-based diagnostics and therapies [239].

Tracer techniques have emerged as indispensable tools in modern ophthalmology, enabling visualization and quantification of physiological and pathological processes at the molecular, cellular, and systemic levels. Though tracer techniques have been discussed in many other fields, such as neurology, oncology, and cardiology, they have not been systematically reviewed in ophthalmology. This review is the first to discuss their mechanism and application in ophthalmology. We clarify the concept of eye–brain axis and address current challenges and future directions of tracing, aiming to guide precision medicine. By utilizing specific molecular probes, the techniques achieve personalized assessment and therapy. Diagnostically, they enable early detection and precise classification. Therapeutically, they enable real-time drug tracking and patient stratification for targeted and radionuclide therapies.

## Figures and Tables

**Table 1:** Comparison of FFA and ICGA in ophthalmic diagnostics.

Technique		FFA	ICGA
**Tracer**		Sodium fluorescein Water-soluble Low molecular weight (376 Da)	Indocyanine green Lipophilic Larger molecular complex (775 Da) Binds to plasma proteins
**Excitation/emission**		~490 nm (blue)/~530 nm (yellow–green)	~805 nm (NIR)/~835 nm (Infrared)
**Tissue penetration**		Limited (primarily retinal vessels)	Strong (choroidal vessels)
**Clinical application**	Diabetic retinopathy	Detects microaneurysms, capillary dropout, and intraretinal microvascular abnormalities [24,25] Identifies neovascularization [24] Assesses blood–retinal barrier integrity [24]	Not typically used
	Age-related macular degeneration	Sensitive for classic CNV [26,27] Assesses leakage [28] Monitors anti-VEGF therapy efficacy [29]	Sensitive for occult CNV [30,31] Gold standard for PCV [32] Guides PDT [33]
	Choroidal tumor	Limited (dye leakage)	**Choroidal melanoma:** Early hypofluorescence, irregular tumor vessels, late leakage [34] **Choroidal nevus:** Well-defined hypofluorescence [34] **Choroidal hemangioma:** Early rapid filling, late hyperfluorescence [35] **Choroidal metastasis:** Vascular dilation or beading [35]
	Idiopathic multifocal choroiditis	CNV activity assessment [36]	Inflammatory activity assessment [36]
	White dot syndrome Stromal choroiditis (VKH syndrome, BRC, sarcoidosis)	Wreath-like lesions (mid phase) Assesses retinal vascular leakage [37] Not typically used	Hypofluorescent spots (mid to late phase) Assesses choroidal perfusion and inflammation [37] Hypofluorescent dark dots (late phases) [38]
	Retinal vascular occlusions	Assesses CME [39]	Not typically used
**Side** **effect**		Nausea Vomiting Allergy	Mild nausea Vomiting Contraindicated if allergic to iodine

FFA: fundus fluorescein angiography. ICGA: indocyanine green angiography. CNV: choroidal neovascularization. PCV: polypoidal choroidal vasculopathy. VEGF: vascular endothelial growth factor. PDT: photodynamic therapy. VKH: Vogt–Koyanagi–Harada. BRC: birdshot retinochoroiditis. CME: cystoid macular edema.

**Table 2:** Advantages and disadvantages of different tracing techniques.

Property	Tracers	Imaging Principle	Spatial Resolution	Cost and Accessibility	Clinical Evidence Level	Pros	Cons	Applications	Refs.
**Optical**	Fluorescent tracer	Excitation by UV/blue/green light; Electron excitation and internal conversion; Photon emission (fluorescence) during return to ground state; Signal detection	High (Cellular/Subcellular level with multiphoton microscopy)	Cost-effective; widely accessible in standard clinics	Routine Clinical Use: Gold standard for retinal and choroidal imaging	Stable signal Cost-effective Low allergenic potential Suitable for vessel imaging and aqueous humor dynamics	Low signal intensity Prolonged tissue retention Photobleaching	Long-term monitoring of hydrogel degradation (dry eye) Visualization of ocular lymphatic system Detection of retinal amyloid and tau Unconventional pathway of aqueous humor tracing Detection of conjunctiva amyloid Visualization of CSF entry into ON (glaucoma) Evaluation of IOP reduction efficiency of nanoformulations Tracking of retinal drug delivery Assessment AMD location, type, and activity	[23,48,94,95,96,97,98,99,100,101,102,103,104,105]
	Photoacoustic tracer	Laser irradiation; Light absorption and heat conversion; Thermal expansion; Ultrasound wave generation and detection	High: Penetrates deep tissue with resolution up to 4.1 μm	High Cost: Requires expensive, specialized imaging systems	Preclinical/Early Clinical: Proven in animal models	Rare photobleaching High spatial resolution and contrast Deep tissue penetration Low detection limit Non-ionizing	Expensive imaging system	Quantitative monitoring of lymphatic drainage In vivo retina imaging Early detection of AMD and diabetic retinopathy Investigation of ocular malignancy biopsies Imaging of Aβ plaques in retina Differentiation of bacterial ocular infections Imaging of retinal/choroidal vessels, RPE, lens, iris	[51,52,53,56,60,106,107,108,109]
**Isotope**	Radioisotope	γ-ray emission during decay; Detection by SPECT/PET camera; Computer-based 2D/3D image reconstruction	Moderate/Low: SPECT typically achieves ~10 mm resolution	High Cost: Approximately $1900 per scan	Clinical Use: Gold standard for systemic staging (e.g., OAL)	Rare allergy Objectivity Digitization Theranostics	High cost Radioactivity Regulatory challenges Uneven availability; Background scattering Absence of interdisciplinary teams	Diagnosis and therapy evaluation of ocular adnexal lymphoma Sarcoidosis Uveal melanoma and hepatic metastasis Ocular and orbital melanoma	[68,110,111,112,113]
	Stable isotope	^13^C-glucose: Replace carbon atoms of tracers with ^13^C; Participation in glycolysis, TCA cycle, pentose phosphate pathway, and anabolic side reactions; Metabolite extraction; LC–MS/MS analysis; Calculation of percent enrichment	Ultra-high: Nanometer-scale resolution in vitro (LC-MS/MS dependent)	High Cost: Requires advanced equipment (LC-MS/MS, NMR)	Preclinical Research: Laboratory use for pathway mapping	Radiation-free Metabolism analysis	Required for advanced equipment	Probing metabolic activity Selection of neuroprotective factor Detection of blood–aqueous barrier permeability Evaluation of drug delivery system Detection of glucose metabolism in lens Calculation of zinc in retina and RPE	[84,114,115,116,117]
Metal	GNP	Covalently or electrostatically attach ligands to GNP surface; Illumination by specific wavelength; Surface plasmon resonance; Absorption and scattering of light at specific wavelengths; Bright spots against dark background	High: Sub-micron (nanoscale, 1–120 nm)	Moderate: Dependent on synthesis/conjugation complexity	Preclinical: Validated in mouse/rabbit models	Size adjustability High drug loading	Cytotoxicity Corona formation	Conjugation of anti-VEGF antibodies in the treatment of B-chronic lymphocytic leukemia Inhibition of corneal and retinal neovascularization	[118,119]
	Gd-based tracer	Potent paramagnetic ion; Shortening the T1 relaxation time of adjacent water protons; T1-hyperintensity; Measurement of enhancement amplitude and spatial distribution	High: MRI-dependent (e.g., 4 cm orbital coil enhances resolution)	High Cost: Standard MRI facility costs	Clinical Utility: Used for assessing specific physiological barriers	Sensitivity in hT2-FLAIR	Gadolinium deposition Difference between the movement of tracers and solutes Influenced by BBB permeability Time-consuming	Reflecting the integrity of blood–retinal barrier Prediction of ON infiltration in RB	[91,120]

UV: ultraviolet. CSF: cerebrospinal fluid. ON: optic nerve. IOP: intraocular pressure. AMD: age-related macular degeneration. SPECT: single-photon-emission computed tomography. PET: positron-emission tomography. RPE: retinal pigment epithelium. TCA cycle: tricarboxylic acid cycle. LC-MS/MS: liquid chromatography with tandem mass spectrometry. GNP: gold nanoparticle. VEGF: vascular endothelial growth factor. Gd: gadolinium. hT2-FLAIR: heavily T2-weighted fluid-attenuated inversion recovery. BBB: blood–brain barrier. RB: retinoblastoma.

**Table 4:** Preclinical and clinical outcomes of key ocular tracers.

Tracer	Application	Preclinical Findings	Clinical Outcomes	Clinical Translation Barriers	Refs.
FFA	Retinal vessel imaging	Early DR rat model Aqueous outflow pathway research in Ad5.myocilinY437H mouse model of glaucoma	Retinal vascular imaging (DR, AMD) Pediatric retinal diseases (retinal vasculopathies, uveitis, disc edema, Coats disease)	Invasiveness Vomiting Unable to image choroidal vessels clearly Lack of standardization: No unified administration protocol	[25,29,219,233]
ICG	Choroidal imaging (PCV, CNV)	Laser-induced CNV rat model Lipid-driven AMD	CSC (151 eyes): asymmetric venous pattern 76.8%; 1 dominant vortex vein 43.7% Gold standard for PCV	Prolonged retinal retention Iodine allergy risk WF-ICGA requires specialized widefield imaging systems	[31,133]
[^18^F]FDG PET/CT	OAL Uveal melanoma Uveitis	Blue light-induced retinal degeneration mouse EIU rat model	Gold standard for OAL staging Predict 1-year survival in metastatic UM	Radiation dose Physiological uptake by periocular structures Low sensitivity for small UM	[70,129,234]
[^123^I]IMP SPECT	UM	Murine melanoma models	UM (19 patients): 24 h-delayed imaging detected all 12 histologically confirmed UM UM (99 patients): 96.3% PPV, 97.2% NPV in untreated cases; useful for atypical diagnoses.	Low spatial resolution (10 mm)	[127,129]
^13^C-glucose	Retinal metabolism (DR, photoreceptor function)	RPEs of aging mice dcKO mouse (HK2/PKM2 double knockout in rod PRs)	Research tool for metabolic flux analysis	Not real-time Technically demanding	[81,82,235]
Gd-based MRI	Aqueous humor dynamics BRB integrity	Rodent glaucoma models Tg-MYOCP370L glaucoma mouse model: Gd signals significantly increased in anterior chamber	Assesses drug effects on aqueous production Distinguishes AEC/PEC barrier dysfunction	IV injection Requires dedicated orbital coil	[93,236]

FFA: sodium fluorescein. AEC: anterior eye chamber. AMD: age-related macular degeneration. BRB: blood–retinal barrier. CNV: choroidal neovascularization. CSC: central serous chorioretinopathy. dcKO: double conditional knockout. DR: diabetic retinopathy. EIU: endotoxin-induced uveitis. FFA: fundus fluorescein angiography/sodium fluorescein. Gd: gadolinium. HK2: hexokinase 2. ICG: indocyanine green. ICGA: indocyanine green angiography. IOP: intraocular pressure. IV: intravenous. MRI: magnetic resonance imaging. OAL: ocular adnexal lymphoma. PCV: polypoidal choroidal vasculopathy. PEC: posterior eye chamber. PKM2: pyruvate kinase M2. PPV: positive predictive value. PR: photoreceptor. RPE: retinal pigment epithelium. SPECT: single-photon-emission computed tomography. UM: uveal melanoma. WF-ICGA: widefield indocyanine green angiography.

## Data Availability

No new data were created or analyzed in this study.
